# Comparative seven year outcomes of RYGB and SADI-S as revisional procedures for weight recurrence regain after sleeve gastrectomy: weight loss trajectory, reflux control, and metabolic safety

**DOI:** 10.1007/s00464-026-12692-x

**Published:** 2026-03-16

**Authors:** Asaad F. Salama, Abdelwahed Yahmadi, Hamzah El Baba, Jawher Baazaoui, Khadija Gibreal, Mohamed Bougmiza, Mohammed Al Kuwari

**Affiliations:** 1 Department of Bariatric Surgery, Hamad General Hospital, Hamad Medical Corporation, Doha, Qatar; 2 Department of General Surgery, Weill Cornell Medicine–Qatar, Doha, Qatar; 3 Department of General Surgery, Hamad General Hospital, Hamad Medical Corporation, Doha, Qatar; 4 Department of Community and Preventive Medicine, Primary Health Care Corporation, Doha, Qatar

**Keywords:** Revisional bariatric surgery, SADI-S, Roux-en-Y gastric bypass, Sleeve gastrectomy failure, Long-term outcomes, Weight regain, Metabolic surgery

## Abstract

**Background:**

Sleeve gastrectomy (SG) is widely performed, yet 20–50% of patients experience insufficient weight loss or weight regain, leading to revisional surgery. Roux-en-Y gastric bypass (RYGB) and single-anastomosis duodeno-ileal bypass (SADI-S) are two commonly used revisional procedures, but long-term comparative data remain limited. This study evaluates 7-year outcomes of RYGB versus SADI-S as revisional surgeries for weight recurrence after SG.

**Methods:**

A retrospective analysis was conducted on all adults undergoing revisional RYGB or SADI-S between 2014 and 2015 after inadequate weight loss or weight recurrence post-SG. Demographic, anthropometric, biochemical, and comorbidity-related variables were assessed at baseline, 1, 5, and 7 years. Statistical analyses included t-tests, chi-square tests, Kaplan–Meier curves, and multivariate regression (significance set at *p* < 0.05).

**Results:**

The cohort included 105 patients (RYGB = 62; SADI-S = 43). SADI-S patients had higher baseline and pre-revision BMI. Across all follow-up points, SADI-S achieved significantly greater %TWL, %EWL, and BMI reduction, demonstrating superior long-term weight-loss durability. RYGB yielded markedly better GERD resolution (95 vs. 5%, *p* = 0.02), while remission of diabetes, hypertension, dyslipidemia, and asthma was similar between groups. Nutritional profiles differed: SADI-S was associated with lower calcium, zinc, folate, and vitamin D levels, whereas RYGB patients had lower vitamin B12. Overall complication rates, including bleeding, marginal ulcer, internal hernia, dumping syndrome, severe malnutrition, and iron-deficiency anemia, were not statistically significant.

**Conclusions:**

Both RYGB and SADI-S are effective and safe revisional options after SG. SADI-S offers superior long-term weight-loss and metabolic outcomes, whereas RYGB remains preferable for patients with significant or persistent GERD. Tailoring revisional procedure selection to patient characteristics and ensuring lifelong nutritional monitoring are essential for optimizing long-term outcomes.

Obesity continues to represent a major global health challenge, with rising prevalence and substantial clinical and economic burden. Bariatric surgery remains the most effective long-term treatment for severe obesity, consistently outperforming nonsurgical interventions in sustained weight reduction and comorbidity improvement. Among surgical options, sleeve gastrectomy (SG) has become the predominant primary procedure due to its technical simplicity, favorable safety profile, and reliable early outcomes [1, 2]. Despite these advantages, 20–50% of SG patients experience inadequate weight loss, significant weight regain, or complications such as severe gastroesophageal reflux disease (GERD), ultimately necessitating revisional surgery [3–5].

Roux-en-Y gastric bypass (RYGB) has long served as the standard revisional procedure, particularly for patients with refractory GERD. More recently, single-anastomosis duodeno-ileal bypass (SADI-S) has gained attention for its technical feasibility and robust metabolic impact [6]. However, while both procedures have become widely adopted, comparative long-term data—especially beyond five years—remain insufficient. This gap is clinically relevant, as obesity is a chronic and relapsing disease, requiring extended or even lifelong follow-up to fully characterize the durability of revisional outcomes [6].

In practice, the choice of revisional procedure is often influenced by the patient’s main complaint. For example, patients who mainly complain of weight recurrence are offered a more malabsorptive procedure; however, those who mainly complain of severe GERD symptoms are usually offered Roux-en-Y gastric bypass. This highlights the importance of evaluating both weight-related and symptom-specific outcomes when comparing revisional strategies [7, 8].

Although both procedures, RYGB and SADI-S, demonstrated effectiveness as revisional procedures, their long-term results regarding weight loss durability, GERD symptom control, and comorbidity resolution beyond five years remain less well defined.

Nutritional safety remains central for the choice of revisional surgery. SADI-S, with its long biliopancreatic limb, raises concerns about micronutrient deficiencies and bone health, whereas RYGB has its own spectrum of nutritional complications. This also raises the need for long-term comparative data to balance efficacy against nutritional safety. Given the increasing use of revisional bariatric surgery and the limited availability of long-term comparative data, this study provides important insights into the differential benefits and risks of RYGB and SADI-S. By integrating 7-year anthropometric, metabolic, and symptom-specific outcomes, this work supports a more individualized and evidence-based approach to procedure selection [7, 9].

## Methods

This retrospective cohort study compared the long-term outcomes in patients undergoing revisional bariatric surgery after insufficient weight loss after sleeve gastrectomy (SG) or weight recurrence. Suboptimal outcomes after sleeve gastrectomy were defined according to accepted international standards as either insufficient weight loss—defined as a percentage of total weight loss (%TWL) of less than 20% and/or a percentage of excess weight loss (%EWL) of less than 50% at 18–24 months postoperatively—or significant weight regain following an initial satisfactory weight loss response [10, 11]. The study included all adult patients who underwent either RYGB or SADI-S at our institution between January 1, 2014, and December 31, 2015. The insufficient weight loss or severe weight recurrence regain was to be revised according to the clinical standards of failed SG. Patients were excluded if they received a revisional operation other than RYGB or SADI-S, if revision was performed solely for non-weight-related complications such as GERD or stricture, or if they did not follow-up adequately after the operation. A total of 105 patients met the inclusion criteria and were included: 62 RYGB and 43 SADI-S patients.

### Surgical technique

All procedures were performed as revisional laparoscopic procedures after SG, and none were converted to open procedures.

*RYGB*: The Roux-en-Y gastric bypass was performed using a standardized technique. A small gastric pouch (30–50 ml) was fashioned by transecting the previously sleeved stomach approximately 4 cm distal to the gastroesophageal junction with additional trimming performed when necessary to optimize pouch configuration. The alimentary (Roux) limb was constructed with a length of 100 cm, and the biliopancreatic limb measured 125 cm. Both Petersen’s space and the mesenteric defect at the jejunojejunostomy were systematically closed to minimize the risk of internal herniation. No intra-abdominal drains were routinely placed.

*SADI-S*: After inspection of the abdominal cavity, a retropyloric-retroduodenal dissection was carried out to expose approximately 3 cm of the duodenum distal to the pylorus, which was then transected. The small bowel length was measured from the ileocecal junction, and a 300-cm ileal common channel was constructed. A double layer end- to- side duodeno-ileal anastomosis was created in an isoperistaltic fashion. No intra-abdominal drains were routinely placed.

Demographic and baseline clinical data were obtained from electronic medical records. The variables included age, sex, height, weight prior to SG, nadir weight after SG, and weight prior to revisional surgery. Comorbidity data were collected and classified as diabetes, hypertension, dyslipidemia, asthma, obstructive sleep apnea, and GERD.

Perioperative variables, including operative time and length of stay, were also extracted. Laboratory markers, including hemoglobin, micronutrients, lipid profile, ferritin, folate, vitamin B12, and vitamin D, were recorded at baseline, 1, 5, and 7 years to assess nutritional status at later time points.

At 1-, 5-, and 7-year post-revision follow-up, anthropometric measurements were obtained. Variables included body weight, BMI, percentage excess weight loss (percent EWL), percentage total weight loss (percent TWL), and body weight reduction.

All complications after the operation, such as bleeding, internal hernia, marginal ulcers, dumping syndrome, severe malnutrition, vitamin deficiencies, and iron deficiency anemia, were carefully reviewed through electronic records from surgery to the date of data collection.

The SPSS version 26 was used to perform statistical analysis. Independent samples based on distributions (either independent-sample t-tests or Mann Whitey U tests). Chi-square test or Fisher exact test was used to analyze categorical variables. Kaplan Meier curves were used to determine time-to-event data (e.g., complication-free survival), whereas Cox proportional hazards techniques were employed to estimate outcome predictors. A *p*-value < 0.05 was considered statistically significant.

## Results

### Baseline characteristics

The study examined 105 individuals who returned to surgery after unsuccessful sleeve gastrectomy to perform a revisional bariatric surgery, 62 underwent conversion to RYGB, and 43 were converted from SG to SADI-S. The age of both groups was similar as the mean age of RYGB was 40.66 years, SD 9.47, and that of SADI-S was 39.35 years, SD 9.28 (*p* = 0.48). But there was significant difference in sex distribution. Females constituted 65.8 percent of the RYGB group and 61.5 percent of the SADI-S group (*p* = 0.01), indicating that there might be gender-related factors that affect the surgical decision-making [4, 5]. Also, SADI-S patients were revised earlier with an average of 3.53 ± 1.29 years after SG as opposed to 4.13 ± 1.31 years in RYGB patients (*p* = 0.02). It could be indicative of a rapid weight recurrence regain after SG in subjects who received SADI-S as a revisional conversion surgery (Table 1).

**Table 1:** Baseline characteristics

Variable	RYGB (n = 62) Mean (SD)	SADI-S (*n* = 43) Mean (SD)	*P*-value
Age (years)	40.66 (9.47)	39.35 (9.28)	0.48
Sex (M/F) *n* (%)			
F	52 (65.8)	27 (34.2)	0.01*
M	10 (38.5)	16 (61.5)	
Years from LSG to revision	4.13 (1.31)	3.53 (1.29)	0.02*
Height (cm)	162.85 (6.91)	165.12 (8.64)	0.14
Weight before LSG (kg)	128.73 (26.95)	141.02 (27.14)	0.02*
BMI before LSG (kg/m^2^)	48.15 (8.25)	50.40 (11.46)	0.24
Lowest weight after LSG (kg)	90.07 (18.00)	101.84 (21.58)	0.005*
Lowest BMI after LSG (kg/m^2^)	33.76 (6.13)	32.92 (13.99)	0.71
Weight before revisional surgery (kg)	107.16 (21.20)	119.05 (23.53)	0.008*
BMI before revisional surgery (kg/m^2^)	40.22 (6.45)	43.51 (7.07)	0.01*
ASA score (I–IV)	2.15 (0.39)	2.12 (0.39)	0.7

*F females, *M* males, *LSG* laparoscopic sleeve gastrectomy, *BMI* body mass index, *ASA* American Society of Anesthesiologists

Anthropometric measurements (Table 1), indicated that SADI-S patients exhibited higher body weight both before SG and prior to revision. Their pre-SG weight was 141.02/27.14 kg on average in comparison to 128.73/26.95 kg in RYGB patients (*p* = 0.02). Pre-revision weight was also significantly higher in the SADI-S group (119.05 + 23.53 kg) compared with the RYGB group (107.16 + 21.20 kg; *p* = 0.008). Accordingly, the SADI-S group had a significantly higher pre-revision BMI (43.51 ± 7.07 kg/m^2^) compared with the RYGB group (40.22 ± 6.45 kg/m^2^; *p* = 0.01) [12–14].

### Pre-operative comorbidities

The RYGB and SADI-S groups showed comparable preoperative comorbidity profiles (Table 2). The prevalence of diabetes, hypertension, dyslipidemia, obstructive sleep apnea, and asthma did not differ significantly between groups. Diabetes was present in 11 RYGB and 7 SADI-S patients, and hypertension in 11 and 8 patients, respectively, with non-significant *p*-values. Although dyslipidemia appeared more frequently in the RYGB group (71.4 vs. 28.6%) (Table 2).

**Table 2:** Co-morbidities pre-op

Variable	RYGB *N* (%)	SADI-S *N* (%)	*P*-value
Diabetes			
No	51 (58.6)	36 (41.4)	0.84
Yes	11 (61.1)	7 (38..9)	
Hypertension			
No	51 (59.3)	35 (40.7)	0.9
Yes	11 (57.9)	8 (42.1)	
Dyslipidaemia			
No	52 (57.1)	39 (42.9)	0.31
Yes	10 (71.4)	4 (28.6)	
OSA			
No	60 (58.3)	43 (41.7)	0.64
Yes	2 (100.0)	0 (0.0)	
Asthma			
No	55 (57.3)	41 (42.7)	0.40
Yes	7 (77.8)	2 (22.2)	
GERD			
No	31 (44.3)	39 (55.7)	0.001*
Yes	31 (88.6)	4 (11.4)	

*OSA obstructive sleep apnea, *GERD* gastro-esophageal reflux disease

The only significant difference in preoperative comorbidities was the markedly higher prevalence of GERD among RYGB patients (88.6 vs. 11.4%, *p* = 0.001). This disparity indicates that GERD was a major determinant in selecting RYGB, consistent with its established efficacy in managing refractory reflux [12]. In contrast, the low prevalence of GERD among SADI-S patients suggests that their revisions were primarily driven by weight-related indications.

### Intraoperative and perioperative data

Intraoperative outcomes (Table 3), did not differ significantly between the RYGB and SADI-S groups, indicating comparable procedural efficiency and perioperative safety. Operative time was similar for both procedures, with RYGB averaging 2:00:45 h and SADI-S 2:03:20 h. Length of hospital stay was likewise comparable (3.69 vs. 3.59 days; *p* = 0.51) (Table 3).

**Table 3:** Intraoperative data

Variable	RYGB (*n* = 62)	SADI-S (*n* = 43)	*p*-value
Operative time (minutes)	2::00:45:00 (0:39–03:05)	2:03:20:93 (0:30–06:65)	0.71
Length of hospital stay (days)	3.69 (0.87)	3.59 (0.74)	0.51

### Anthropometric outcomes

During the 7-year follow-up, SADI-S consistently achieved superior weight-loss outcomes compared with RYGB (Table 4). Although absolute weight and BMI were similar between groups at each assessment point, SADI-S produced significantly greater reductions in %EWL (53.75 vs. 43.46%, *p* = 0.05), %TWL (21.63 vs. 15.03%, *p* = 0.001), and BMI (9.54 vs. 6.07, *p* < 0.001) at 1 year (Table 4).

**Table 4:** Anthropometric outcomes comparison of RYGB and SADI-S

	RYGB (*n* = 62)	SADI-S (*n* = 43)	*P*-value
Weight at 1 year	89.16 (17.99)	93.45 (18.11)	0.30
BMI at 1 year	33.73 (5.69)	34.09 (6.12)	0.76
%EWL at 1 year	43.46 (27.49)	53.75 (22.83)	0.05
%TWL at 1 year	15.03 (8.43)	21.63 (8.25)	0.000
BMI reduction at 1 year	6.07 (3.44)	9.54 (4.08)	0.000
Weight at 5 years	92.15 (19.27)	93.07 (15.58)	0.79
BMI at 5 years	34.67 (6.00)	34.34 (5.85)	0.78
%EWL at 5 years	34.94 (36.46)	49.67 (30.13)	0.03
%TWL at 5 years	12.49 (10.66)	20.52 (12.09)	0.001
BMI reduction at 5 years	5.13 (4.69)	9.27 (6.28)	0.000
Weight at 7 years	92.17 (21.05)	94.89 (16.49)	0.49
BMI at 7 years	34.64 (6.80)	34.73 (5.52)	0.94
%EWL at 7 years	37.53 (41.24)	46.67 (29.36)	0.23
%TWL at 7 years	13.23 (12.28)	19.59 (11.97)	0.01
BMI reduction at 7 years	5.44 (4.96)	8.94 (6.08)	0.002

**Fig. 1:**
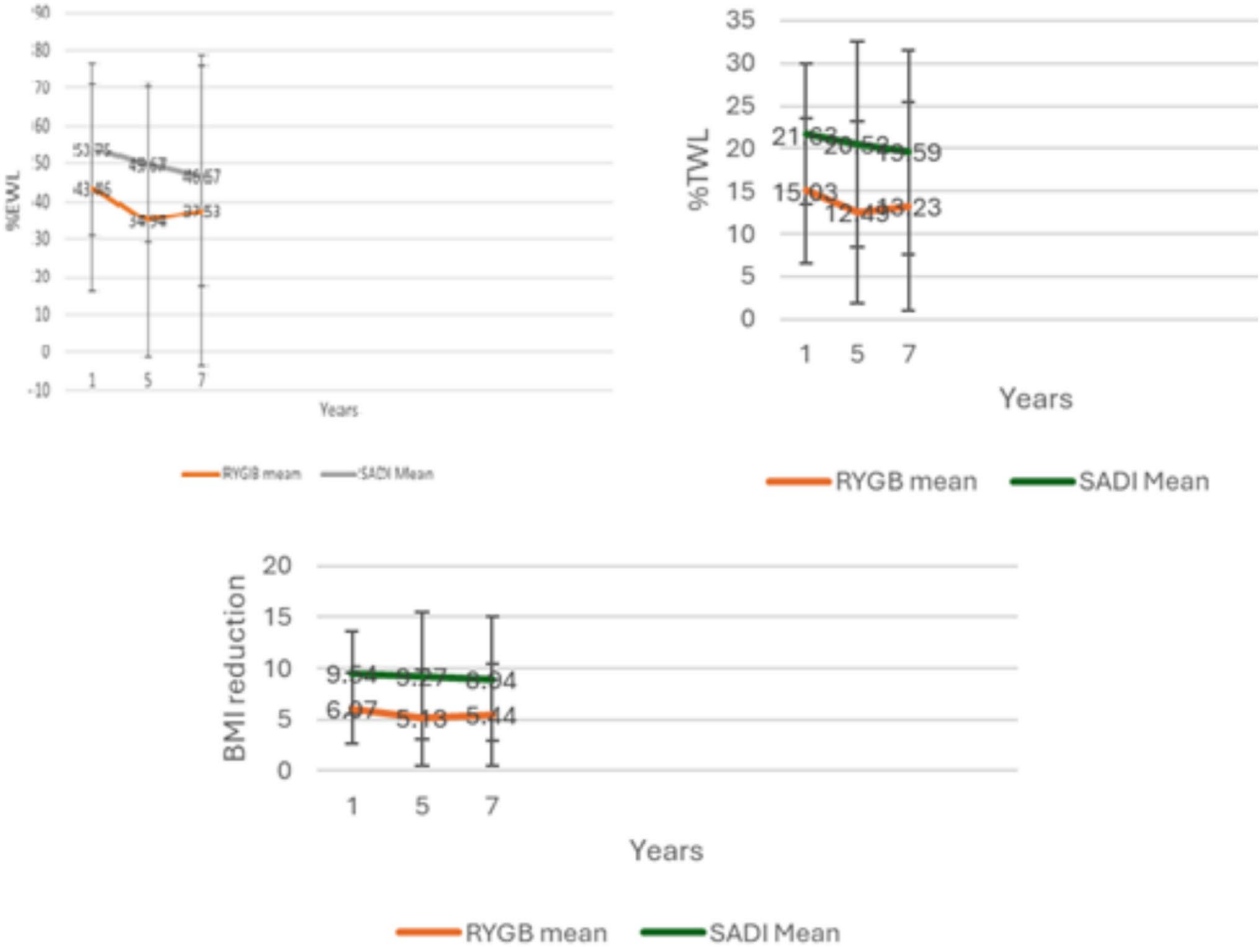
Comparative long-term weight loss trajectories after revisional RYGB and SADI-S. Mean percentage excess weight loss (%EWL), percentage total weight loss (%TWL), and body mass index (BMI) reduction are reported at 1, 5, and 7 years after revisional surgery. SADI-S consistently showed higher %EWL and %TWL and greater BMI reduction than RYGB across all follow-up intervals. Values are reported as means with standard deviation. RYGB is shown in orange and SADI-S in green (Color figure online)

These advantages persisted long-term. At 5 years, SADI-S maintained higher %EWL (49.67 vs. 34.94%, *p* = 0.03), %TWL (20.52 vs. 12.49%, *p* = 0.001), and BMI reduction (9.27 vs. 5.13, *p* < 0.001). By 7 years, absolute weight and BMI were comparable, yet SADI-S continued to show superior %TWL (19.59 vs. 13.23%, *p* = 0.01) and BMI reduction (8.94 vs. 5.44, *p* = 0.002). These findings are clearly depicted in Fig. 1.

Collectively, these findings indicate that SADI-S provides more durable and clinically meaningful weight-loss outcomes than RYGB as a revisional surgery after inadequate weight loss following sleeve gastrectomy.

### Postoperative laboratory outcomes

Postoperative laboratory assessments (Table 5), at 1, 5, and 7 years demonstrated distinct differences in malabsorption-related parameters between the RYGB and SADI-S groups. Hemoglobin levels remained comparable across all time points. In contrast, calcium levels were consistently lower in the SADI-S group at 5 and 7 years (*p* = 0.00 and *p* = 0.04) (Table 5).

**Table 5:** Post-op lab tests (1, 5, and 7 years) comparison of RYGB and SADI-S

	RYGB (*n* = 62)	SADI-S (*n* = 43)	*P*-Value
Hb_1year	11.91 (1.82)	11.71 (2.54)	0.77
Hb_5year	11.78 (1.64)	12.07 (2.07)	0.43
Hb_7year	11.91 (1.77)	11.78 (1.31)	0.70
Cholesterol_1year	4.87 (1.40)	4.05 (0.80)	0.002*
Cholesterol_5year	4.51 (0.93)	4.08 (0.77)	0.02*
Cholesterol_7year	4.65 (0.85)	4.21 (0.71)	0.01*
TG_1year	1.04 (0.49)	0.92 (0.53)	0.28
TG_5year	1.06 (0.52)	0.77 (0.24)	0.001*
TG_7year	0.99 (0.47)	0.77 (0.28)	0.006*
HDL_1year	1.51 (0.37)	1.28 (0.29)	0.001*
HDL_5year	1.55 (0.44)	1.33 (0.28)	0.007*
HDL_7year	1.59 (0.48)	1.42 (0.27)	0.03*
LDL_Calc_1year	2.67 (0.63)	2.34 (0.72)	0.02*
LDL_Calc_5year	2.50 (0.77)	2.39 (0.60)	0.49
LDL_Calc_7year	2.58 (0.69)	2.43 (0.62)	0.27
Iron_1year	10.32 (5.86)	12.7 (6.32)	0.09
Iron_5year	9.29 (4.87)	11.51 (6.15)	0.10
Iron_7year	11.41 (5.96)	10.77 (5.32)	0.65
HbA1C_1year	5.28 (0.60)	5.08 (0.40)	0.08
HbA1C_5year	5.52 (0.65)	5.29 (0.53)	0.08
HbA1C_7year	5.52 (0.71)	5.48 (0.69)	0.78
Copper_1year	17.99 (4.83)	15.98 (4.38)	0.22
Copper_5year	19.10 (3.70)	18.21 (5.05)	0.59
Copper_7year	17.73 (7.87)	17.01 (3.93)	0.80
Zink_1year	10.83 (2.48)	9.41 (2.36)	0.02*
Zink_5year	11.35 (2.19)	9.94 (1.53)	0.01*
Zink_7year	10.61 (1.73)	9.51 (1.30)	0.04*
Vit D_1year	19.49 (10.98)	13.90 (7.43)	0.005*
Vit D_5year	27.92 (23.10)	16.43 (10.59)	0.02*
Vit D_7year	23.79 (13.80)	22.11 (10.09)	0.52
Ferritin_1year	63.66 (149.49)	57.15 (64.66)	0.83
Ferritin_5year	108.19 (203.02)	91.47 (184.71)	0.71
Ferritin_7year	104.15 (238.01)	75.51 (120.47)	0.55
Folate_1year	24.70 (11.59)	13.20 (7.71)	0.00*
Folate_5year	28.42 (12.18)	16.52 (12.09)	0.01*
Folate_7year	27.81 (13.88)	14.22 (9.96)	0.00*
Vit B12_1year	260.69 (136.87)	386.53 (282.82)	0.01*
Vit B12_5year	209.19 (115.39)	405.54 (329.00)	0.02*
Vit B12_7year	256.21 (140.88)	465.25 (285.78)	0.00*

Lipid profiles showed more pronounced differences. SADI-S patients exhibited significantly lower total cholesterol at 1, 5, and 7 years (*p* = 0.002, 0.02, and 0.01), and lower triglycerides at 5 and 7 years (*p* = 0.001 and 0.006), alongside reduced HDL and LDL at all assessments, with LDL reaching statistical significance at 1 year.

Micronutrient evaluation revealed higher zinc and folate levels in RYGB patients. In contrast, vitamin B12 concentrations were consistently higher after SADI-S.

### Comorbidity outcomes

Comorbidity outcomes (Table 6) were largely comparable between RYGB and SADI-S, with no statistically significant differences for type 2 diabetes, hypertension, dyslipidemia, or asthma.

**Table 6:** Comorbidity outcomes comparison of RYGB versus SADI after sleeve gastrectomy

Variable	RYGB *N* (%)	SADI-S *N* (%)	*p*-value*
Diabetes			
Complete remission or improved	4 (44.4)	5 (55.6)	0.24
No changes/worse	6 (85.7)	1 (14.3)	
Hypertension			
Complete remission or improved	0 (0.0)	2 (100.0)	0.38
No changes/worse	8 (61.5)	5 (38.5)	
Dyslipidemia			
Complete remission or improved	6 (66..7)	3 (33.3)	0.99
No changes/worse	2 (50.0)	2 (50.0)	
Asthma			
Complete remission or improved	2 (100.0)	0 (0.0)	0.99
No change/worse	2 (66.7)	1 (33.3)	
GERD			
Complete remission or improved	19 (95.0)	1 (5.0)	0.02*

Diabetes remission or improvement occurred in 44.4% of RYGB and 55.6% of SADI-S patients (*p* = 0.24). Hypertension outcomes were similar overall, with improvement observed only in the SADI-S group (Table 6).

Dyslipidemia remission or improvement was 66.7% in RYGB and 33.3% in SADI-S (*p* = 0.99).

Asthma improvement rates were low and did not differ between procedures.

In contrast, GERD outcomes favored RYGB: complete remission or improvement occurred in 95.0 versus 5.0% with SADI-S (*p* = 0.02), and persistent or progressive GERD was more common after SADI-S.

### Postoperative complications

Postoperative complications (Table 7) were uncommon in both groups, and no statistically significant differences were observed between RYGB and SADI-S. Bleeding occurred in one RYGB patient and in none of the SADI-S patients (*p* = 0.99). Marginal ulceration was reported more frequently after RYGB, though not significantly so (*p* = 0.61). Internal hernia occurred only in the RYGB group (two cases), but this difference was also not significant (*p* = 0.64). Dumping syndrome was more frequently reported after RYGB (81.8 vs. 18.2%), although the difference did not reach statistical significance (*p* = 0.19) (Table 7).

**Table 7:** Comparison of complications outcomes following RYGB versus SADI-S

Variable	RYGB n (%)	SADI-S n (%)	*p*-value
Bleeding			
Yes	1 (100)	0 (0.0)	0.99
No	61 (58.7)	43 (41.3)	
Marginal ulcer			
Yes	4 (80.0)	1 (25.0)	0.61
No	58 (58.0)	42 (42.0)	
Internal Hernia			
Yes	2 (100.0)	0 (0.0)	0.64
No	60 (58.3)	43 (41.7)	
Dumping syndrome			
Yes	9 (81.8)	2 (18.2)	0.19
No	53 (56.4)	41 (43.6)	
Vitamin deficiencies			
Yes	18 (62.1)	11 (37.9)	0.69
No	44 (57.9)	32 (42.1)	
Severe malnutrition			
Yes	1 (33.3)	2 (66.7)	0.74
No	61 (59.8)	41 (40.2)	
IDA			
Yes	27 (55.1)	22 (44.9)	0.44
No	35 (52.5)	21 37.5)	

*IDA iron deficiency anemia

Nutritional and metabolic complications showed similar patterns between procedures. Vitamin deficiencies were observed in 62.1% of RYGB and 37.9% of SADI-S patients (*p* = 0.69). Severe malnutrition was rare, occurring in one RYGB and two SADI-S patients (*p* = 0.74). Iron-deficiency anemia was common in both cohorts—55.1% in RYGB and 44.9% in SADI-S—with no significant difference (*p* = 0.44).

Notably, neither group experienced anastomotic leak, bowel obstruction, or stricture.

## Discussion

### Long-term weight loss outcomes

Current evidence indicates that both Roux-en-Y gastric bypass (RYGB) and single-anastomosis duodeno-ileal bypass (SADI-S) are effective revisional procedures following inadequate weight loss after sleeve gastrectomy. However, SADI-S consistently demonstrates superior long-term weight-loss outcomes. Patients undergoing SADI-S achieve significantly greater percentages of excess weight loss (EWL), total weight loss (TWL), and BMI reduction at 1, 5, and 7 years, reflecting more durable weight-loss maintenance [3, 6].

This enhanced effect is likely attributable to the greater malabsorptive component of SADI-S, driven by duodenal diversion and a longer bypassed intestinal segment, which augments long-term metabolic and caloric malabsorption. Such characteristics may be particularly advantageous in patients with higher pre-revision BMI or early failure after sleeve gastrectomy. By contrast, although RYGB produces meaningful weight loss, its mixed restrictive-malabsorptive mechanism may offer comparatively less sustained benefit in higher-BMI revisional populations [6, 13, 15].

### Resolution of obesity-related comorbidities

Both revisional procedures achieved comparable improvement or remission of obesity-related comorbidities—including type 2 diabetes, hypertension, dyslipidemia, and asthma—with no statistically significant differences between RYGB and SADI-S. These findings suggest that weight loss itself is the principal driver of metabolic improvement before and after revision, regardless of the surgical technique [12, 13].

However, the outcomes for gastroesophageal reflux disease (GERD) differed markedly between procedures. RYGB resulted in significantly higher rates of GERD remission or improvement, consistent with its well-established anti-reflux mechanism that diverts both gastric acid and bile from the esophagus. These results reaffirm RYGB as the preferred revisional option for patients presenting with severe or persistent GERD after sleeve gastrectomy [16].

### Perioperative and short-term safety outcomes

Operative time and length of hospital stay did not differ significantly between the groups, indicating comparable perioperative efficiency and safety for both RYGB and SADI-S. These findings support the feasibility of performing either procedure as a revisional intervention in experienced bariatric centers. The similarity in early postoperative recovery further reinforces SADI-S as a viable revisional procedure [6, 14].

Early postoperative complications were also comparable. Neither cohort experienced anastomotic leak, bowel obstruction, or stricture, and no significant differences were observed in other early surgical events. These results underscore that, with appropriate patient selection and surgical expertise, revisional bariatric procedures can be performed with low perioperative risk despite the inherent complexity of re-operative surgery [13, 17].

### Nutritional and metabolic outcomes

Long-term laboratory follow-up showed that most nutritional parameters—including hemoglobin, albumin, and total protein—remained comparable between the two groups throughout the study period. In contrast, differences emerged in lipid profiles and selected micronutrients. SADI-S patients demonstrated greater reductions in total cholesterol and triglycerides over time, reflecting the more pronounced metabolic effects of this procedure [3, 5, 18, 19].

Both procedures were associated with persistent micronutrient deficiencies, particularly in iron, vitamin D, zinc, folate, and vitamin B12. Although severe malnutrition was rare, these findings underscore the need for lifelong nutritional monitoring after revisional surgery. The greater malabsorptive component of SADI-S, in particular, necessitates strict adherence to postoperative supplementation to mitigate long-term deficiency risks [6, 20, 21].

### Postoperative complications and long-term safety

Overall complication rates were low and did not differ significantly between RYGB and SADI-S, with no meaningful variation in bleeding, marginal ulceration, internal hernia, dumping syndrome, or iron-deficiency anemia. These findings indicate that both procedures share comparable safety profiles when performed in appropriately selected patients.

Severe malnutrition was uncommon in both cohorts, further supporting the long-term safety of these revisional procedures. However, the persistence of micronutrient deficiencies underscores the chronic nutritional risks inherent to malabsorptive procedures. These results highlight the importance of structured postoperative follow-up and strict adherence to supplementation to sustain long-term outcomes after revision [5, 22].

### Clinical implications and procedure selection

This study demonstrates that RYGB and SADI-S serve complementary roles rather than interchangeable ones in revisional bariatric surgery. SADI-S offers superior long-term weight-loss durability and metabolic benefits, making it particularly suitable for patients with higher-BMI or early post-sleeve gastrectomy failure. In contrast, RYGB offers a clear advantage in managing GERD and is the preferred option for patients presenting with reflux-dominant symptoms [3, 5, 23].

Accordingly, selection of the revisional procedure should be individualized, integrating factors such as baseline BMI, presence of GERD, nutritional risk, and the patient’s capacity for long-term follow-up. Shared decision-making between the surgeon and the patient remains essential for optimizing outcomes and minimizing postoperative complications [16, 22].

## Conclusions

This 7-year comparative analysis demonstrates that both Roux-en-Y gastric bypass and single-anastomosis duodeno-ileal bypass are effective and safe revisional procedures for inadequate weight loss after sleeve gastrectomy. Both operations produced durable long-term weight reduction with acceptable complication rates when supported by structured follow-up and nutritional monitoring.

However, clinically meaningful differences were evident. SADI-S yielded superior long-term weight-loss outcomes, reflected in higher total and excess weight loss and greater BMI reduction at 1, 5, and 7 years. These findings highlight the stronger malabsorptive effect of SADI-S, particularly benefiting patients with higher pre-revisional weight or significant weight regain after sleeve gastrectomy.

In contrast, RYGB demonstrated a clear advantage in managing gastroesophageal reflux disease, achieving substantially higher rates of symptom resolution. Outcomes related to other obesity-associated comorbidities, including diabetes and hypertension, were similar between procedures. Although nutritional parameters differed, severe malnutrition and major surgical complications remained infrequent in both groups.

Overall, these results support a personalized approach to revisional bariatric surgery, balancing long-term weight-loss goals, symptom control, and nutritional risk. Lifelong follow-up remains essential to optimize outcomes and sustain the benefits of either revisional strategy.

## Strengths and limitations

### Strengths

- *Long-term follow-up*: The study provides 7-year outcomes, offering rare long-term comparative data on RYGB and SADI-S as revisional procedures—a time horizon seldom reported in the literature.
- *Direct comparison of two major revisional techniques*: The analysis contrasts two of the most commonly performed revisional operations after failed sleeve gastrectomy, enabling meaningful clinical comparisons in weight-loss durability, metabolic outcomes, and safety.
- *Comprehensive clinical assessment*: Anthropometric, biochemical, metabolic, and comorbidity-related data were systematically collected at multiple time points (baseline, 1, 5, and 7 years), strengthening the robustness of outcome evaluation.
- *Real-world clinical relevance*: The study reflects actual surgical practice in a high-volume bariatric center, enhancing the external validity and applicability of the findings to similar settings.
- *Balanced safety analysis*: Detailed reporting of both surgical and nutritional complications provides a holistic evaluation of long-term risks associated with each revisional procedure.

### Limitations

- *Retrospective design*: The retrospective nature limits control over confounders, introduces potential selection bias, and restricts the ability to establish causality.
- *Single-center study*: Findings may not be fully generalizable to other institutions with different patient populations, surgical expertise, or postoperative protocols.
- *Non-randomized allocation:* Procedure selection was influenced by clinical presentation (e.g., GERD severity, baseline BMI), creating inherent baseline differences between groups that may affect comparative outcomes despite statistical adjustments.
- *Relatively small sample size, especially for subgroup analyses*: Although adequate for primary outcomes, the sample limits the statistical power for detecting differences in less frequent complications or comorbidity-specific outcomes.
- *Incomplete long-term nutritional data*: While several laboratory markers were assessed, variability in follow-up adherence may have limited the completeness of micronutrient and metabolic assessments at later time points.
- *Absence of patient-reported outcomes*: Measures such as quality-of-life, symptom burden, and satisfaction were not evaluated, limiting insight into functional or psychosocial impact.
